# A Treatise upon the Diseases and Hygiène of the Organs of the Voice

**Published:** 1845-06

**Authors:** 


*A Treatise upon the Diseases and Hygiène of the Organs of the Voice.* By Colombat de l'Isere, Chevalier of the Order of the Legion of Honor, Doctor of Medicine, Founder of the Orthophonic Institute of Paris for the treatment of all vices of speech, Diseases of the Voice, &c. Translated by J. F. W Lane, M. D. 24mo. pp. 220. Boston, 1845.

We have carefully looked over this translation, but can discover no adequate reason—unless it be a labour of love—which has led the translator to give it to the profession. He states, in his preface, that "it was undertaken at the request of an eminent Professor of Elocution, with the hope that it might prove useful to the general reader, by pointing out to him the physiology and diseases of the organs of the voice, the medical treatment of the more common of these affections, and the conditions necessary to preserve them in health."—So that the production is not intended for the profession, but for the laity, and scarcely, therefore, demands notice from us.

We wish that Dr. Lane had declined the request of the "eminent Professor"—unless, indeed, he had added—what we believe him competent to do—information that is scantily, or, not at all, afforded by the French author. The Anatomy is certainly defective; the physiology in many respects questionable; the pathological portion common-place, and the hygiène trite,—occupying sixteen of the small pages. The strictly surgical portion has been omitted by Dr. Lane, for a somewhat singular reason—"in order to render the work as compact as possible!" and for another, which may, by the way, apply to other portions of the "Treatise"—"because the surgeon, whom these points alone concerns, will find them given at length in the surgical works of the day."

The main subjects treated of are—"the description of the vocal instrument,"—"the voice and its formation,"—"the mechanism of the voice,"—"the pharyngean voice or *faucette*,"—the diseases, and the hygiène of the voice.

The *Falsette* or *Falsetto voice* is doubtless familiar to our readers. It is generally supposed to be produced in the pharynx, although Savart refers it to the ventricles of the larynx. It is the *voce di testa* of singers, in contradistinction to the *voce di petto* or *laryngeal notes*, or *notes of the first register*. Its precise mechanism, however, has not been demonstrated. We refer to it at present, merely to state, that M. Colombat objects to the word *falsette*, because there is nothing *false* in the notes; and substitutes *faucette*, to indicate that they are produced in the *fauces*. Should it be shown, however, that they are not pharyngeal, the substitution of one word for the other would convey a most erroneous idea.

The Translator appears to have executed his work creditably. He has, however, been somewhat inattentive in regard to proper names, some of which have been greatly *estropiés*. For example, we scarcely recognized our old acquaintances, Savart, Ettmuller, Lenhossek, Liscovius, Grenié, Piorry, Gerdy, and others. Again, Dr. Lane may admit the force of example in employing "*autopsy*" for "*cadaveric autopsy.*" Autopsy has, however, a distinct meaning, and we regret that it should ever be employed in any other than its legitimate acceptation,—of "ocular demonstration," or "attentive examination by one's self." This new version was introduced about the same time with the translation of the French word *observation* by the English word "observation," instead of "case;" as "*observation first*," "*observation second.*" This we are glad to find is almost abandoned; and it was probably a blunder made originally by some one, who was not aware of the usual acceptation of the term, and afterwards copied by others, who ought to have known better. Its import is either "observation" or "case," according to circumstances.

